# Impact of mask use on face recognition: an eye-tracking study

**DOI:** 10.1186/s41235-022-00382-w

**Published:** 2022-04-08

**Authors:** Janet Hui-wen Hsiao, Weiyan Liao, Ricky Van Yip Tso

**Affiliations:** 1 Department of Psychology, University of Hong Kong, Pokfulam Road, Hong Kong, Hong Kong SAR China; 2 The State Key Laboratory of Brain and Cognitive Sciences, University of Hong Kong, Hong Kong, Hong Kong SAR China; 3 Department of Psychology, The Education University of Hong Kong, Tai Po, New Territories, Hong Kong SAR China; 4 Psychological Assessment and Clinical Research Unit, The Education University of Hong Kong, Tai Po, New Territories, Hong Kong SAR China

**Keywords:** Face recognition, Face mask use, Eye movement, EMHMM

## Abstract

We examined how mask use affects performance and eye movements in face recognition and whether strategy change reflected in eye movements is associated with performance change. Eighty-eight participants performed face recognition with masked faces either during learning only, during recognition only, or during both learning and recognition. As compared with the baseline condition where faces were unmasked during both learning and recognition, participants had impaired performance in all three scenarios, with larger impairment when mask conditions during learning and recognition did not match. When recognizing unmasked faces, whether the faces were learned with or without a mask on did not change eye movement behavior. Nevertheless, when recognizing unmasked faces that were learned with a mask on, participants who adopted more eyes-focused patterns had less performance impairment as compared with the baseline condition. When recognizing masked faces, participants had more eyes-focused patterns and more consistent gaze transition behavior than recognizing unmasked faces regardless of whether the faces were learned with or without a mask on. Nevertheless, when recognizing masked faces that were learned without a mask, participants whose gaze transition behavior was more consistent had less performance impairment as compared with the baseline condition. Thus, although eye movements during recognition were mainly driven by the mask condition during recognition but not that during learning, those who adjusted their strategy according to the mask condition difference between learning and recognition had better performance. This finding has important implications for identifying populations vulnerable to the impact of mask use and potential remedial strategies.

## Significance statement

The abrupt change in social life due to preventive mask use during the COVID-19 pandemic calls for urgent research on its impact on social cognition, in particular the recognition of faces, as well as identification of vulnerable populations and possible remedial strategies. While individual differences in face recognition have been much studied in recent years, there has not been any research on how mask use influences individuals’ face recognition memory, and whether some people are more vulnerable to the impact than others. Here, we show that the most challenging scenario of mask use on face recognition is when the mask conditions during face learning and recognition do not match, including recognizing unmasked faces that were masked during learning, or recognizing masked faces that were unmasked during learning. In these scenarios, individuals with better abilities to adjust their eye movement strategy according to the mask condition difference are affected less by mask use in recognition performance. These findings suggest that individuals with poor cognitive flexibility to switch strategies during face learning and recognition, or poor problem-solving skills to develop a new visual routine for recognizing masked faces, can become vulnerable to the impact of mask use on face recognition ability during daily life. Potential vulnerable populations include children, older adults, and individuals with autism spectrum disorders. Explicit instructions on better eye movement strategies to tackle these scenarios may be beneficial to them. After the world has recovered from the pandemic, these strategies can continue being used under face covering scenarios.

## Introduction

The COVID-19 pandemic has completely changed our social life: Social distancing has become the new normal, and protective mask use has become prevalent. This situation is likely to last for a long time. Nevertheless, there is virtually no research on how widespread mask use in a society impacts how people remember and recognize faces, and whether a particular information processing strategy can facilitate face recognition when masks are used. Face recognition is an essential skill in social life. Misidentification of faces may interfere with normal social functioning and enhance social anxiety (e.g., Davis et al., 2011). Thus, it may affect mental health at the individual level. Also, face recognition ability is shown to be associated with social biases in the society (e.g., Morgan & Hills, 2019; Young et al., 2011). Thus, its impact on human life is far reaching and there is an urgency to fill this research gap.

Facial features such as the eyes, the nose, and the mouth convey important information for face recognition. When people wear masks, some facial features, i.e., the nose and the mouth, are covered and not available for memory encoding (face learning) or retrieval (face recognition). Mask wearing is shown to impair holistic face processing (Freud et al., 2020), an important perceptual skill supporting face perception and recognition (e.g., Tanaka & Simonyi, 2016; Wang et al., 2012). It is also shown to impair face matching performance (Carragher & Hancock, 2020; Dhamecha et al., 2014), even for super-recognizers (Noyes et al., 2021). In face recognition memory, mask wearing can happen during face learning, during face recognition, or during both face learning and recognition. When the information available for memory encoding during face learning and memory retrieval during face recognition does not match, such as recognizing an unmasked face that was learned with a mask on, or recognizing a masked face that was learned without a mask, recognition can become particularly challenging. Indeed, a mismatch between information used for memory encoding and retrieval has been consistently reported to impair memory recall performance (e.g., Uner & Roediger, 2018; Unsworth et al., 2020). Thus, we may need to change our information extraction strategy in response to the mask conditions during face learning and recognition in order to reduce the impact of mask use on our recognition performance. Nevertheless, it remains unclear how we change our information extraction strategy when we remember or recognize masked faces, and whether a particular strategy can facilitate face recognition when masks are used. Here, we aimed to fill this research gap through examining how mask use under different scenarios affects recognition performance and eye movement behavior during face recognition. We also aimed to examine whether information extraction strategy change in response to mask use, as reflected in eye movement behavior, is associated with recognition performance change.

Recent research has reported substantial individual differences in eye movement patterns during face recognition that can indicate differences in recognition performance and cognitive abilities (e.g., Chan et al., 2018; Chuk et al., 2017b; Hsiao et al., 2021a; Peterson & Eckstein, 2013; Peterson et al., 2016). To take individual differences in both temporal and spatial dimensions of eye movements into account in data analysis, Chuk et al. (2014) developed a machine learning-based approach, eye movement analysis with hidden Markov models (EMHMM; hidden Markov model, or HMM, is a type of time-series statistical model in machine learning), which provides quantitative measures of eye movement pattern and consistency (Chan et al., 2018; Hsiao et al., 2021b). In this approach, an individual’s eye movement pattern in a visual task is first summarized using an HMM, including person-specific regions of interest (ROIs) and transition probabilities among the ROIs. Individual HMMs can be clustered according to similarities (Coviello et al., 2014) to discover representative eye movement patterns in the population, and similarities among individual eye movement patterns can be quantified using their data likelihood given the models of the representative patterns. In addition, consistency of an individual’s eye movements across task trials can be directly measured as the entropy of the HMM (entropy is a measure of predictability; higher entropy indicates lower consistency; Cover & Thomas, 2006).

Using EMHMM on eye movement data during face recognition, two representative eye movement patterns, i.e., eyes-focused and nose-focused patterns, have been consistently reported in adult face recognition, and the eyes-focused pattern is shown to be associated with better recognition performance (An & Hsiao, 2021; Chuk et al., 2017a, 2017b; Hsiao et al., 2021a). These eye movement patterns for face recognition observed in adults have been shown to be consistent over time within an individual and impervious to the influence of transitory mood changes (An & Hsiao, 2021; Hsiao et al., 2021a; Peterson & Eckstein, 2013; Peterson et al., 2016). This phenomenon may be related to adults’ abundant experience in face recognition, which leads to a well-developed, consistent visual routine for recognizing faces that is inflexible to change. Indeed, using EMHMM, Hsiao et al. (2020) showed that adults had more consistent (lower entropy) eye movement patterns than children in face recognition. This finding is consistent with the observation in the literature that adult face recognition performance has limited plasticity for improvement through training, which may also be related to adults’ abundant face recognition experience that has made their recognition performance reach a capacity limit (Tree et al., 2017).

The findings summarized above suggest that when encountering faces with a mask, adults’ information extraction strategy for either face learning or recognition may be affected by their well-developed visual routines for recognizing unmasked faces, and individuals who have a better ability to develop a new routine according to the current mask condition of the faces without being affected by the old routine may perform better in face recognition when masks are used. For example, when recognizing an unmasked face that was learned with a mask on, only information not covered by the mask, including the eye and forehead region, was available for memory encoding. Thus, during recognition, adopting a more eyes- or forehead-focused eye movement pattern than one’s original visual routine may be beneficial. Similarly, when recognizing a masked face, only information in the eye and forehead region is available for retrieval. Hence, it may be beneficial to focus on developing new strategies based on the information available without being affected by one’s original routine for recognizing unmasked faces, especially when the face was learned without a mask on and thus not all information available during memory encoding is available for recognition. Note however that some previous studies have suggested a dissociation between eye movement patterns during face learning and face recognition (Chuk et al., 2017a; Henderson et al., 2005). These findings suggested that our original, well-developed visual routines during face recognition may still play an important role for successful recognition when encountering masked faces. Thus, it remains unclear what eye movement strategies are beneficial for learning and recognizing masked faces.

Accordingly, here we examined three scenarios where face recognition performance could be impaired by mask use: when masks were used during face learning only, during recognition only, or during both face learning and recognition. In the baseline condition, unmasked faces were used during both face learning and recognition. We hypothesized that as compared with the baseline condition, mask use impaired face recognition performance in all three scenarios, and larger impairment may be observed when there was a mismatch in mask condition between face learning and face recognition. We also hypothesized that when recognizing an unmasked face that was learned with a mask on, individuals may differ in how well they could adjust their eye movement strategy to focus on the available information during learning, i.e., the eye and forehead region. And those who had a larger change in eye movement pattern toward the eye and forehead region may have better performance. In the scenario of recognizing a masked face, those who were able to develop a new, consistent visual routine unaffected by the old routine for unmasked faces, especially when the face was learned without a mask on, may have better recognition performance. To test these hypotheses, we conducted an eye tracking study of face recognition under these scenarios and use the EMHMM method to quantify participants’ eye movement pattern and consistency in different scenarios. Recent research has suggested that general intelligence is a poor predictor for face recognition performance (e.g., Wilmer, 2017). However, eye movement behavior in face recognition has been reported to be associated with some cognitive abilities, particularly those related to executive function, visual attention, and working memory (e.g., Chan et al., 2018; Hsiao et al., 2020). To understand whether the hypothesized associations between eye movement behavior change and performance impairment due to mask use could still be observed after partialling out these general intelligence and cognitive ability factors, here we also measured participants’ general intelligence and cognitive abilities.

## Method

### Design

The face recognition task consisted of two phases: a learning phase and a recognition phase. For the learning phase data, we focused our analysis on difference in eye movement behavior between viewing unmasked and masked faces. The design consisted of a within-participant variable mask condition (unmasked vs. masked), and the dependent variables were eye movement measures. For the recognition phase data, the design consisted of two within-participant variables: mask condition during learning (unmasked vs. masked) and mask condition during recognition (unmasked vs. masked). The dependent variables were recognition performance and eye movement measures (Table 1). Recognition performance was measured in discrimination sensitivity *d*′ and RT. The eye movement measures of interest included eye movement pattern, marginal entropy of the first fixation, conditional entropy of the second fixation, and conditional entropy of the third fixation, as measured using eye movement analysis with hidden Markov models (EMHMM; see the “Eye movement data analysis” section). We then used paired *t* test to examine the impact of mask use in the following three scenarios using the scenario with unmasked faces at both phases as the baseline: (1) Effect of mask use during learning: masked faces at learning and unmasked faces at recognition versus baseline; (2) effect of mask use during recognition: unmasked faces at learning and masked faces at recognition versus baseline; and (3) effect of mask use in the face recognition task: masked faces at both phases versus baseline. Correlation analysis was used to examine whether participants’ recognition performance impairment due to mask use in the above three scenarios was associated with the corresponding change in eye movement behavior. In addition, partial correlation was used to examine whether these associations could be still observed after participants’ general intelligence and cognitive ability measures (including working memory, executive planning, and selective attention) were partialled out.

**Table 1:** Four mask conditions used in the design

		Learning phase	
		Unmasked	Masked
*Recognition phase*	Unmasked	Learn unmasked faces, recognize unmasked faces	Learn masked faces, recognize unmasked faces
	Masked	Learn unmasked faces, recognize masked faces	Learn masked faces, recognize masked faces

### Participant

Participants were 88 university students or graduates (51 females). Their age ranged from 17 to 30 (*M* = 21.1, SD = 2.36). All participants had normal vision or corrected to normal vision by contact lenses or glasses. According to a power analysis, a sample size of 85 was needed to acquire a medium effect size (${f}^{2}$ = .15) in a linear multiple regression with 4 tested predictors (*β* = .2; *α* = .05).

### Materials

#### Face stimuli

The face stimuli consisted of 256 colored frontal-view Asian face images from face databases developed in Professor William Hayward’s lab and the Attention Brain and Cognition Lab, respectively, at the University of Hong Kong (Chan et al., 2018; Chuk et al., 2014; Hsiao et al., 2021a). Half of them were young adult faces, one-fourth were old adult faces, and one-fourth were child faces. Within each face age group, half of the images were females. All faces had a neutral expression and were unfamiliar to the participants. The face images were cropped along the face shape so that only the inner features were available for recognition. Each face subtended a horizontal visual angle of 6° under the viewing distance of 55 cm, equivalent to the size of a real face under a functional distance for face identification (~ 2 m; McKone, 2009). All face images were scaled and aligned according to the distance between the centers of the two eyes.

The face images were randomly divided into two groups to be used as the target and foil images in the recognition task, with the number of faces in each gender by age combination matched between the two groups. For each face identity, images with different mask conditions (i.e., masked and unmasked), lighting conditions (i.e., yellow light and white light), and mask colors (blue mask and white mask) were created using Adobe Photoshop. During the face recognition task, images used during the learning and the recognition phases differed in both lighting condition and mask color for masked faces.

#### Raven’s standard progressive matrices (RSPM)

The RSPM was used to assess participants’ general intelligence (Raven, 2000). It is a multiple-choice test for assessing abstract thinking and reasoning. Participants were required to select a missing piece to complete a matrix-like pattern. The nine-item version, which is derived from the full set of RSPM and has obtained good accuracy in predicting the total score of the full set (Bilker et al., 2012), was used in this study.

#### Two-back task

Two-back tasks were used to assess participants’ working memory ability, i.e., to encode, maintain and update incoming information (Jaeggi et al., 2010). In the verbal two-back task, in each trial participants were presented with a single-digit number at the center of the screen for 1000 ms, followed by a blank screen for 2500 ms. They were asked to judge whether the presented number was the same as the one presented two trials back. In the spatial two-back task, in each trial participants were presented with a symbol appearing at one of 12 possible locations on the screen for 1000 ms, followed by a 2500 ms blank screen. They were asked to judge whether the presented symbol location was the same as the one presented two trials back (Lau et al., 2015). In both tasks, participants finished two blocks of 26 trials each. Accuracy and response times (RTs) of correct trials were measured.

#### Tower of London (TOL) task

The TOL task was used to assess participants’ executive planning ability (Berg & Byrd, 2002). Participants were presented with three color beads randomly placed on three pegs as a starting position together with a goal position. They were asked to move one bead at a time to reach the goal position with the least number of moves. In total, there were 12 trials. We measured number of correct trials, preplanning time (time before the first move) and execution times (time after the first move).

#### Flanker task

The flanker task was used to assess selective attention and response inhibition (Eriksen & Eriksen, 1974; Ridderinkhof et al., 1999). In each trial, participants judged the direction of an arrow flanked by four other arrows. In congruent trials, the flanking arrows pointed in the same direction as the target arrow, whereas in incongruent trials, they pointed in the opposite direction. The stimulus was presented for 500 ms, followed by a blank screen until response. Under a viewing distance of 50 cm, the flanking arrows subtended a horizontal and vertical visual angle of 1.03° × 1.03°, and the target arrow subtended a horizontal and vertical visual angle of .83° × .83°. In total there were 120 trials. We measured the flanker effect as $\frac{\left(I - C\right)}{\left(I + C\right)}$, where *I* and *C* stand for the performance in the incongruent and congruent trials, respectively. Accuracy and correct RTs were measured.

#### Apparatus

Participants’ eye movements during the face recognition task were recorded using an EyeLink 1000 eye tracker. A chinrest was used to minimize head movements. Pupil and corneal reflection tracking mode was used with a sampling rate of 1000 Hz. EyeLink default settings for cognitive research were used for data acquisition, i.e., saccade motion threshold of .1 degree of visual angle, saccade acceleration threshold of 8000 degree/square second, and saccade velocity threshold of 30 degrees.

### Procedure

In the learning phase of the face recognition task, participants were presented with 16 face images, one at a time, and asked to remember the faces. In the recognition phase, participants were presented with the 16 target (old) faces together with 16 foil (new) faces one at a time in a random order and asked to judge whether they had seen the face during the learning phase. The face image stayed on the screen until response. Participants performed eight blocks of the task. To examine the effect of mask use on recognition performance, in each block, among the 16 target faces, there were four stimuli in each of the following conditions: unmasked at both phases, masked at both phases, unmasked at learning and masked at recognition, and masked at learning and unmasked at recognition (Table 1). The 16 foil faces used in the recognition phase matched the conditions of the target faces. The faces used in the four mask conditions were counterbalanced across participants using a Latin square design. Among the eight blocks, participants performed four blocks with faces under white light with blue masks during learning and under yellow light with white masks during recognition; in the other four blocks, the light and mask colors in the two phases were swapped. The light and mask colors used for each face stimulus were counterbalanced across participants. For each participant, the block order was randomized.

Before each learning and recognition phase, a nine-point calibration procedure was performed. Each trial started with a solid circle at the screen center for drift correction. Re-calibration took place whenever drift correction error exceeded 1° of visual angle. During the learning phase, in each trial participants were presented with a face at one of the four quadrants of the screen for 5 s and were asked to remember the face. During the recognition phase, in each trial participants were presented with a face at one of the four quadrants of the screen and were asked to judge whether the face was an old face presented during the learning phase by pressing the “f” key for “yes” responses and “j” key for “no” responses using the left and right index fingers, respectively. The face remained on the screen until they responded. Participants took a short break between blocks. After they finished the face recognition task, they completed the verbal two-back task, the spatial two-back task, the Flanker task, and the TOL task.

### Eye movement data analysis

The eye movement analysis with hidden Markov models (EMHMM) method was used to analyze eye movement data (Chuk et al., 2014). For eye movement data during the learning phase, for each participant, we used one hidden Markov model (HMM) to summarize the participant’s eye movement pattern when viewing unmasked faces, and another HMM to summarize the participant’s eye movement pattern when viewing masked faces. In other words, each participant had two HMMs, with each corresponding to eye movement pattern for unmasked and masked faces, respectively. The variational Bayesian expectation–maximization (VBEM) algorithm (Bishop, 2006) was used to train individual HMMs, and the optimal number of ROIs for each HMM was determined by VBEM from a preset range 1 to 10. (In previous EMHMM studies of face recognition, the median number of ROIs discovered in individual models was typically between 2 and 4 ROIs; e.g., An & Hsiao, 2021; Chan et al., 2018; Chuk et al., 2017a, 2017b; Hsiao et al., 2021a.) Following previous EMHMM studies on face processing, we clustered all individual HMMs to discover two representative eye movement patterns for face learning among the participants, Pattern *A* and Pattern *B*, using the variational hierarchical expectation–maximization (VHEM) algorithm (Coviello et al., 2014). The median of the number of ROIs among the individual HMMs was used to generate the HMMs of the two representative patterns. For each participant, we quantified the eye movement pattern during face learning, for viewing unmasked and masked faces separately, along the dimension contrasting Pattern *A* and Pattern *B* using *A*–*B* scale, which is defined as:

$${(}A - B{)}/(\left| A \right| + |B|)$$

where *A* refers to the log-likelihood of the participant’s eye movement data being generated by the HMM of Pattern *A*, and *B* refers to the log-likelihood of the participant’s data being generated by the HMM of pattern *B* (see, e.g., An & Hsiao, 2021; Chan et al., 2018, 2020a, 2020b, 2020c; Chan et al., 2022a, b; Chuk et al., 2020; Hsiao et al., 2021a, 2021c; Lee et al., 2021; Zhang et al., 2019; Zheng et al., 2022). *A* more positive *A*–*B* scale indicates greater similarity to Pattern *A*, whereas a more negative *A*–*B* scale indicates greater similarity to Pattern *B*.

For eye movement data during the recognition phase, each participant had four HMMs, with each summarizing the eye movement pattern in one of the four mask conditions in Table 1. Similar to the analysis for face learning, we clustered all individual models to discover two representative patterns, Pattern *A* and Pattern *B* and then quantified each participant’s eye movement pattern in each of the four mask conditions using *A*–*B* scale.

In addition to *A*–*B* scale, we examined participants’ eye movement consistency across trials in different mask conditions using the entropy of the HMMs. Entropy is a measure of predictability; higher entropy indicates lower consistency (Cover & Thomas, 2006). In EMHMM, entropy of HMMs has been used to quantify participants’ eye movement consistency during visual tasks (Hsiao et al., 2021b). Previous studies on face recognition have suggested that the first 2–3 fixations in a trial play a more important role in accounting for recognition performance than later fixations (Chuk et al., 2017b; Hsiao & Cottrell, 2008). To better understand the temporal dynamics of eye movement consistency in different mask conditions, we measured marginal entropy of the first fixation, conditional entropy of the second fixation given the first fixation, and conditional entropy of the third fixation given the second fixation, to quantify consistency of the first fixation location, consistency of the transition from the first fixation to the second fixation, and consistency of the transition from the second fixation to the third fixation, respectively (Hsiao et al., 2021b). These three types of entropy were measured separately for each mask condition outlined in Table 1.

## Results

### Face recognition performance

**Fig. 1:**
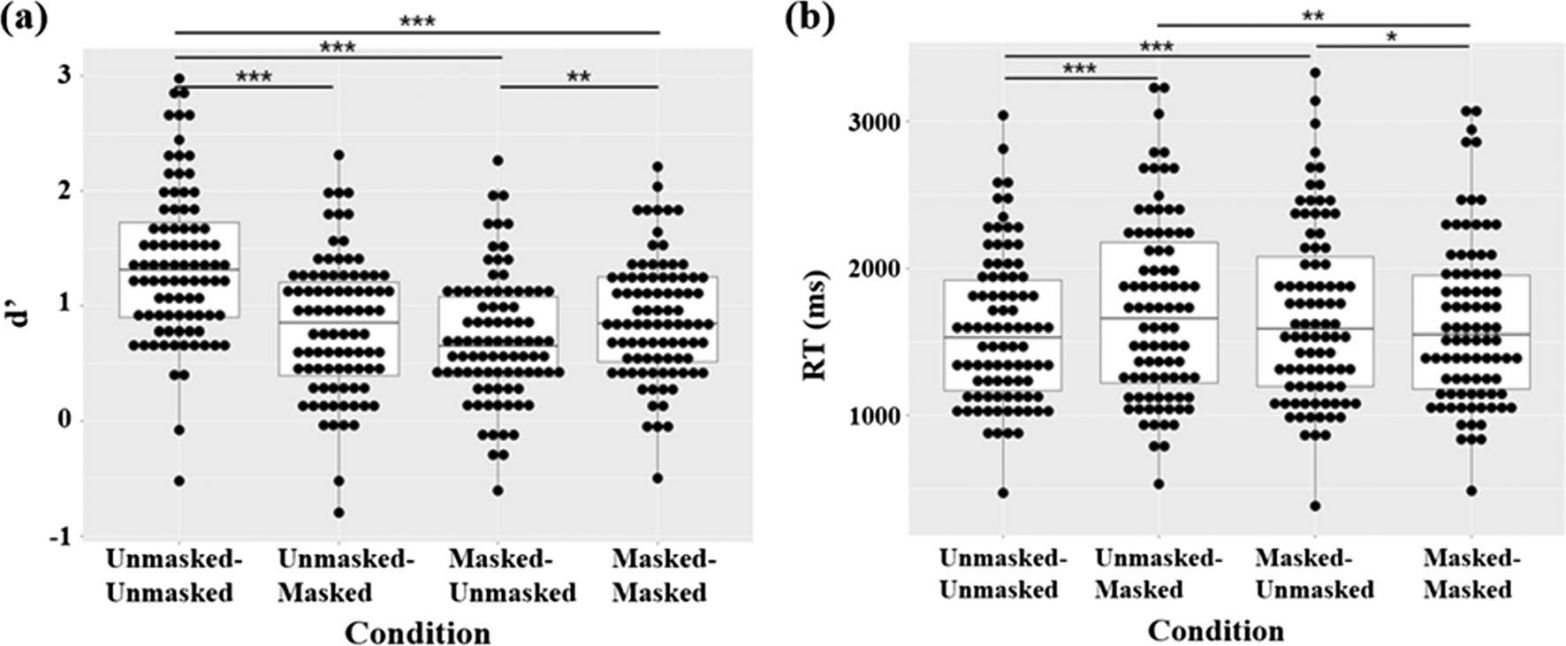
**a** Participants’ *d*′ in different mask conditions (unmasked–unmasked: Faces were unmasked during both learning and recognition. Unmasked–masked: Faces were unmasked during learning and masked during recognition. Masked–unmasked: Faces were masked during learning and unmasked during recognition. Masked–masked: Faces were masked during both learning and recognition). **b** Participants’ RT in ms in different mask conditions (*p < .05, ** *p* < .01, ***p < .001, paired *t* test)

Participants’ face recognition performance as measured in *d*′ and RT is shown in Fig. 1. The 2 × 2 ANOVA showed a main effect of mask condition during learning, *F*(1,87) = 40.110, *p* < .001, ${\eta }_{p}^{2}$ = .316, 90% CI = [.1848, .4268]^1^: Participants had lower *d*′ in the recognition of faces learned with than without a mask on; a main effect of mask condition during recognition, *F*(1,87) = 22.340, *p* < .001, ${\eta }_{p}^{2}$ = .204, 90% CI = [.0900, .3193]: Participants had lower *d*′ when recognizing masked faces than unmasked faces. There was also an interaction effect between mask condition during learning and mask condition during recognition, *F*(1,87) = 63.266, *p* < .001, ${\eta }_{p}^{2}$ = .421, 90% CI = [.2890, .5218]: After learning unmasked faces, participants had lower *d*′ when recognizing them as masked faces than as unmasked faces, *t*(171) =  − 9.103, *p* < .001, *d* =  − .686, 95% CI [− .8504, − .5220]; in contrast, after learning masked faces, participants had lower *d*′ when recognizing them as unmasked faces than as masked, *t*(171) = 2.895, *p* = .022, *d* = .218, 95% CI [.0687, .3677]. These results showed that participants had lower *d*′ when mask conditions during the learning and recognition phases did not match.

In RT, the 2 × 2 ANOVA showed a main effect of mask condition during recognition, *F*(1,87) = 5.343, *p* = .023, ${\eta }_{p}^{2}$ = .058, 90% CI = [.0042, .1508]: Participants had longer RT when recognizing masked faces than unmasked faces; it interacted with mask condition during learning, *F*(1,87) = 26.534, *p* < .001, ${\eta }_{p}^{2}$ = .234, 90% CI = [.1133, .3487]: After learning unmasked faces, participants had longer RT when recognizing them as masked faces than as unmasked faces, *t*(172) = 5.39, *p* < .001, *d* = .406, 95% CI [.2526, .5600]; in contrast, after learning masked faces, participants did not have significantly different RT when recognizing them as unmasked faces or as masked faces, *t*(172) =  − 2.32, *p* = .098.

We then examined the changes in performance due to mask in the three planned comparisons separately. On the effect of mask use during learning (masked–unmasked vs. unmasked–unmasked), participants had lower *d*′, *t*(87) =  − 8.880, *p* < .001, *d* =  − .947, 95% CI [− 1.1980, − .6952], and longer RT, *t*(87) = 4.347, *p* < .001, *d* = .463, 95% CI [.2435, .6833], when recognizing an unmasked face learned with than without a mask on.

On the effect of mask use during recognition (unmasked–masked vs. unmasked–unmasked), participants had lower *d*′, *t*(87) =  − 8.370, *p* < .001, *d* =  − .892, 95% CI [− 1.1393, − .6452], and longer RT, *t*(87) = 5.091, *p* < .001, *d* = .543, 95% CI [.3189, .7665], when recognizing masked faces than unmasked faces that were learned without a mask on.

On the effect of mask use in the face recognition task (masked–masked vs. unmasked–unmasked), participants had lower *d*′, *t*(87) =  − 7.539, *p* < .001, *d* =  − .804, 95% CI [− 1.0440, − .5633], when performing the task with masked faces than with unmasked faces. However, their RT did not differ significantly between the two conditions, *t*(87) = 1.897, *p* = .061.

### Eye movement behavior during face learning

**Fig. 2:**
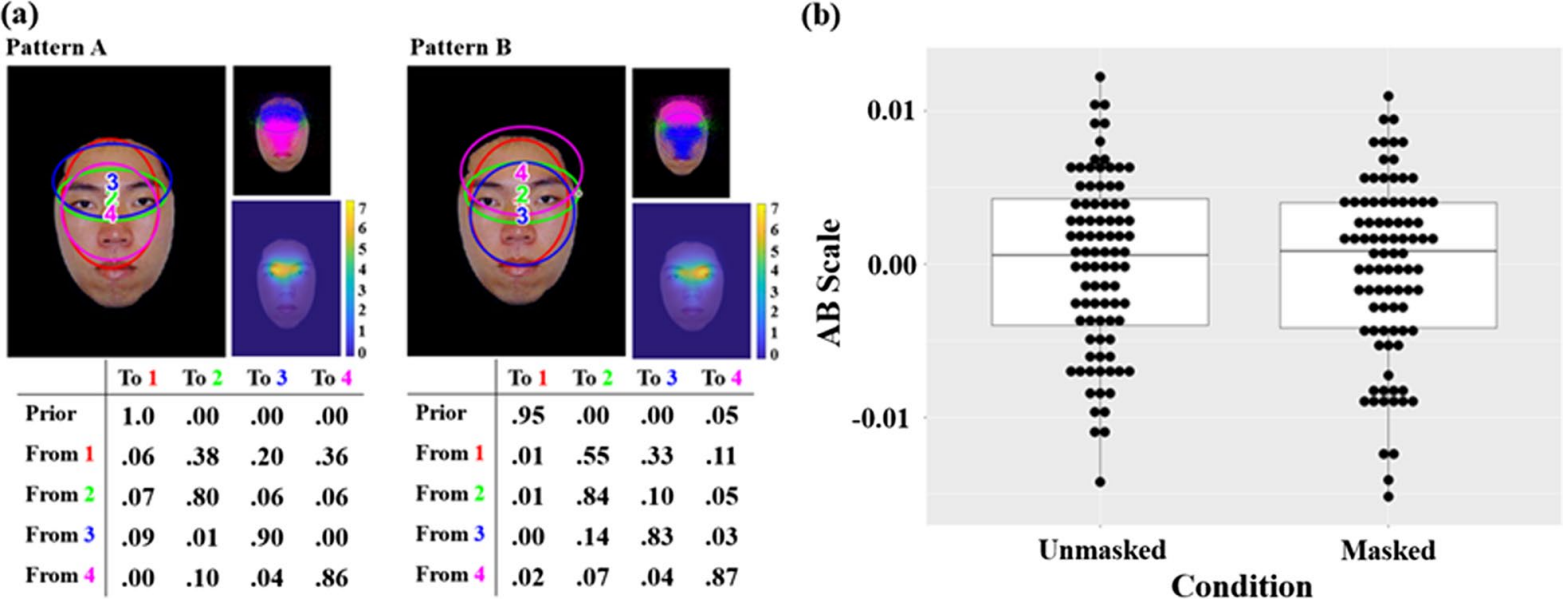
Eye movement data during face learning. **a** The two representative eye movement patterns discovered using EMHMM during face learning: In each pattern, ellipses show ROIs as 2-D Gaussian emissions. Priors in the table show the probabilities that a fixation sequence starts from the ellipse. The table also shows transition probabilities among the ROIs. The smaller image on the top-right shows the assignment of actual fixations to different ROIs. The assignment of fixations to the ROIs was based on the ROI sequence with the largest posterior probability given the fixation sequence. The smaller image on the bottom-right shows the corresponding heatmap. **b** Eye movement pattern measured in *A*–*B* scale in the unmasked and masked conditions during face learning

The two representative eye movement patterns during face learning discovered through clustering using EMHMM are shown in Fig. 2. In Pattern *A*, a scan path always started with a fixation at a broad region centered at the mid-point between the two eyes, covering both the eye region and the nose region (red, 100%). Afterward, it either stayed exploring in the broad region (Red to Magenta, 36%), switched to the eye region (red to green, 38%), or the forehead region (Red to Blue, 20%), and then most likely remained in the same region. In contrast, in Pattern *B*, a scan path typically started with a fixation at a broad region centered at the mid-point between the two eyes (red, 95%), with a small probability to start with a fixation at the forehead region (magenta, 5%). After a fixation at the broad region (red), it most likely switched to the eye region (red to green, 55%), or the nose region (red to blue, 33%), and occasionally to the forehead region (red to magenta, 11%). As compared with Pattern *A*, Pattern *B* also had more transitions between the eyes (green) and the nose (blue) regions, and a larger and higher forehead ROI (magenta). The two patterns significantly differed according to KL divergence estimates (Chuk et al., 2014): Data from participants adopting Pattern *A* were more likely to be generated by Pattern *A* HMM than Pattern *B* HMM, and vice versa for data from participants adopting Pattern *B*, *F*(1, 174) = 316.99, *p* < .001, ${\eta }_{p}^{2}$ = .646, 90% CI [.5776, .6957].

We then examined whether participants’ eye movement behavior, including eye movement pattern and consistency, differed when viewing masked versus unmasked face during face learning. Participants’ eye movement pattern was quantified using *A*–*B* scale according to the two representative patterns, and eye movement consistency was assessed using entropy of the HMMs. The results showed that there was no significant difference in eye movement behavior between viewing masked and unmasked faces during face learning as measured in *A*–*B* scale, *t*(87) =  − .4245, *p* = .672, marginal entropy of the first fixation, *t*(87) = .1793, *p* = .858, conditional entropy of the second fixation given the first fixation, *t*(87) = .7774, *p* = .439, or conditional entropy of the third fixation given the second fixation, *t*(87) = .0997, *p* = .921. In other words, participants’ eye movement behavior did not differ significantly when viewing masked versus unmasked faces during face learning.

### Eye movement patterns during face recognition

**Fig. 3:**
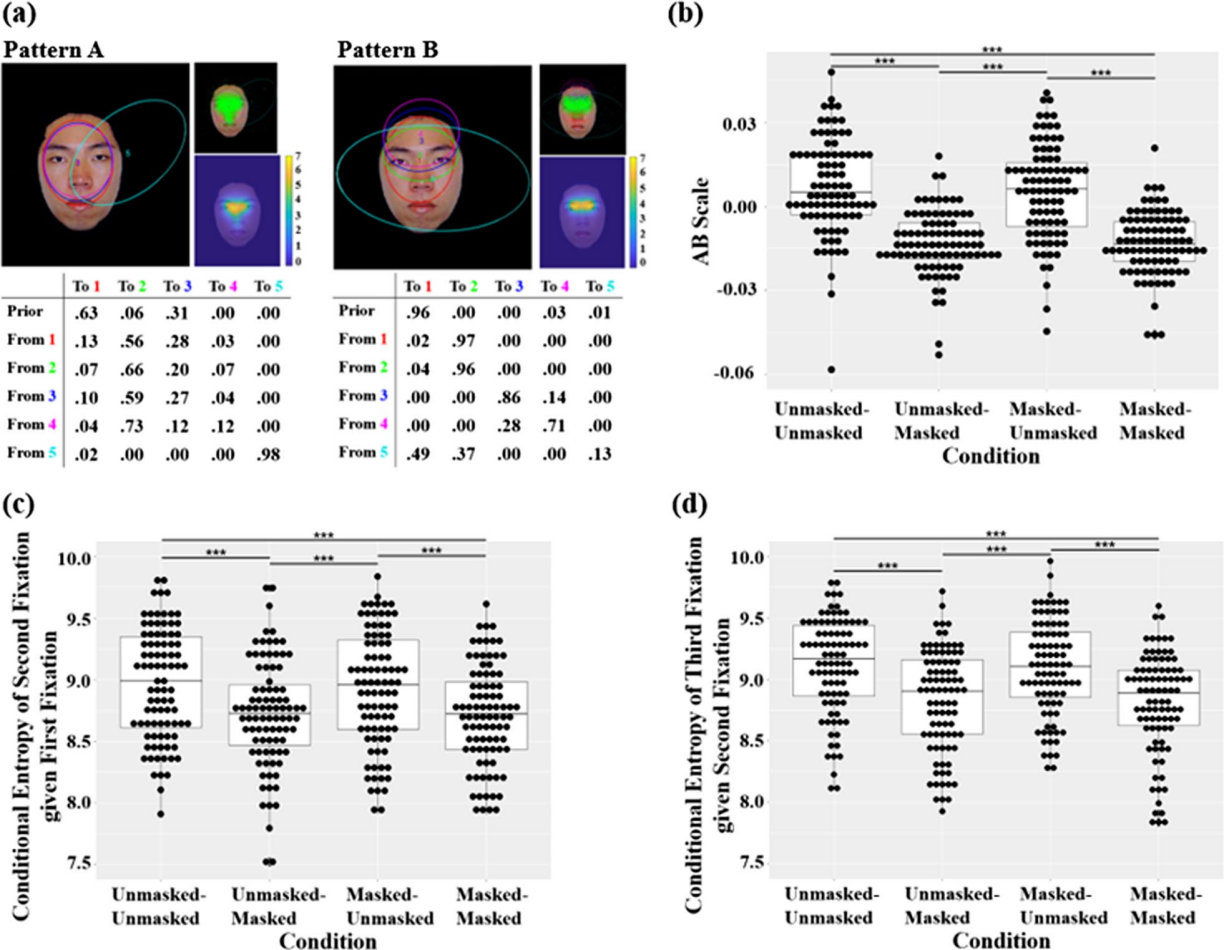
Eye movement data during the recognition phase. **a** The two representative eye movement patterns discovered using EMHMM during face recognition: In each pattern, the ellipses show ROIs as 2-D Gaussian emissions. Priors in the table show the probabilities that a fixation sequence starts from the ellipse. The table also shows transition probabilities among the ROIs. The smaller image on the top-right shows the assignment of actual fixations to different ROIs. The assignment of fixations to the ROIs was based on the ROI sequence with the largest posterior probability given the fixation sequence. The smaller image on the bottom-right shows the corresponding heatmap. Note that ROI 5 (Cyan) in both patterns captures outlier fixations that do not belong to other ROIs. **b** Eye movement pattern measured in *A*–*B* scale in different mask conditions. **c** Eye gaze transition consistency from the first fixation to the second fixation as measured in conditional entropy. **d** Eye gaze transition consistency from the second fixation to the third fixation as measured in conditional entropy (*p < .05, ** *p* < .01, ***p < .001, paired *t* test)

The two representative eye movement patterns during face recognition discovered through clustering using EMHMM are shown in Fig. 3a. In Pattern *A*, participants fixated across a broad region centered at the bridge of the nose between the two eyes, covering both the eye region and the nose region. In contrast, in Pattern *B*, a scan path typically started with a fixation at a broad region centered at the mid-point between the two eyes covering both the eye and the nose regions (red, 96%). Then, it most likely switched to the eye region (red to green, 97%), and then remained in the same region (green to green, 96%). Occasionally, it started (magenta, 3%) and stayed at the forehead region (blue and magenta). The two patterns significantly differed, as data from participants adopting Pattern *A* were more likely to be generated by Pattern *A* HMM than Pattern *B* HMM, and vice versa for data from participants adopting Pattern *B* (following Chuk et al., 2014), *F*(1, 350) = 25.8, *p* < .001, ${\eta }_{p}^{2}$ = .069, 90% CI [.0318, .1146].

We then examined whether participants’ eye movement behavior, including eye movement pattern and consistency, differed among different mask conditions during face learning and recognition (Table 1). The 2 (mask condition during learning) × 2 (mask condition during recognition) ANOVA on eye movement pattern as measured in *A*–*B* scale showed a main effect of mask condition during recognition (Fig. 3b), *F*(1, 87) = 208.24, *p* < .001, ${\eta }_{p}^{2}$ = .705, 90% CI [.6167, .7611]: Participants showed an eye movement pattern more similar to Pattern *A* when recognizing unmasked faces than masked faces. A main effect of mask condition during recognition was also observed in conditional entropy of the second fixation given the first fixation (Fig. 3c), *F*(1, 87) = 68.714, *p* < .001, ${\eta }_{p}^{2}$ = .441, 90% CI [.3103, .5395]: Participants showed more consistent second fixation given the first fixation when recognizing masked faces than unmasked faces. Similarly, a main effect of mask condition during recognition was observed in conditional entropy of the third fixation given the second fixation (Fig. 3d), *F*(1, 87) = 122.552, *p* < .001, ${\eta }_{p}^{2}$ = .585, 90% CI [.4709, .6616]: Participants showed more consistent third fixation given the second fixation when recognizing masked faces than unmasked faces. No main effect or interaction was observed in marginal entropy of the first fixation. Together these results showed that participants eye movements were more eyes-focused (Pattern *B*) with more consistent gaze transition patterns when recognizing masked faces than unmasked faces, regardless of whether the faces were learned with or without a mask on during learning.

We then examined eye movement behavior change due to mask use in the three planned comparisons separately. On the effect of mask use during learning (masked–unmasked vs. unmasked–unmasked), no significant effect was observed in A–B scale, *t*(87) =  − 1.815, *p* = .073, marginal entropy of the first fixation, *t*(87) =  − 1.420, *p* = .159, conditional entropy of the second fixation given the first fixation, *t*(87) =  − 1.108, *p* = .271, or conditional entropy of the third fixation given the second fixation between the two conditions, *t*(87) =  − .715, *p* = .476. These results suggested that eye movement behavior during recognition was mainly driven by information available at the recognition phase but not that presented at the learning phase.

On the effect of mask use during recognition (unmasked–masked vs. unmasked–unmasked), participants had lower *A*–*B* scale, *t*(87) =  − 14.203, *p* < .001, *d* =  − 1.514, 95% CI [− 1.8201, − 1.2080], lower conditional entropy of the second fixation given the first fixation, *t*(87) =  − 6.587, *p* < .001, *d* =  − .702, 95% CI [− .9355, − .4689], and lower conditional entropy of the third fixation given the second fixation, *t*(87) =  − 7.984, *p* < .001, *d* =  − .851, 95% CI [− 1.0950, − .6073], when recognizing masked faces than unmasked faces. No significant difference was observed in marginal entropy of the first fixation, *t*(87) =  − 1.338, *p* = .185. Thus, they had more eye-focused eye movement pattern and more consistent gaze transition behavior when recognizing masked faces than unmasked faces.

On the effect of mask use in the face recognition task (masked–masked vs. unmasked–unmasked), participants had lower *A*–*B* scale, *t*(87) =  − 13.405, *p* < .001, *d* =  − 1.429, 95% CI [− 1.7260, − 1.1320], lower conditional entropy of the second fixation given the third fixation, *t*(87) =  − 6.733, *p* < .001, *d* =  − .718, 95% CI [− .9520, − .4834], and lower conditional entropy of the third fixation given the second fixation, *t*(87) =  − 10.075, *p* < .001, *d* =  − 1.074, 95% CI [− 1.3364, − .8117], when performed the face recognition task with masked faces than with unmasked faces. No significant different was found in marginal entropy of the first fixation, *t*(87) =  − .752, *p* = .454. Thus, they had more eye-focused eye movement pattern and more consistent gaze transition behavior in the recognition task with masked faces than unmasked faces.

### Relationship between eye movement behavior change and performance change due to mask use during face recognition

**Fig. 4:**
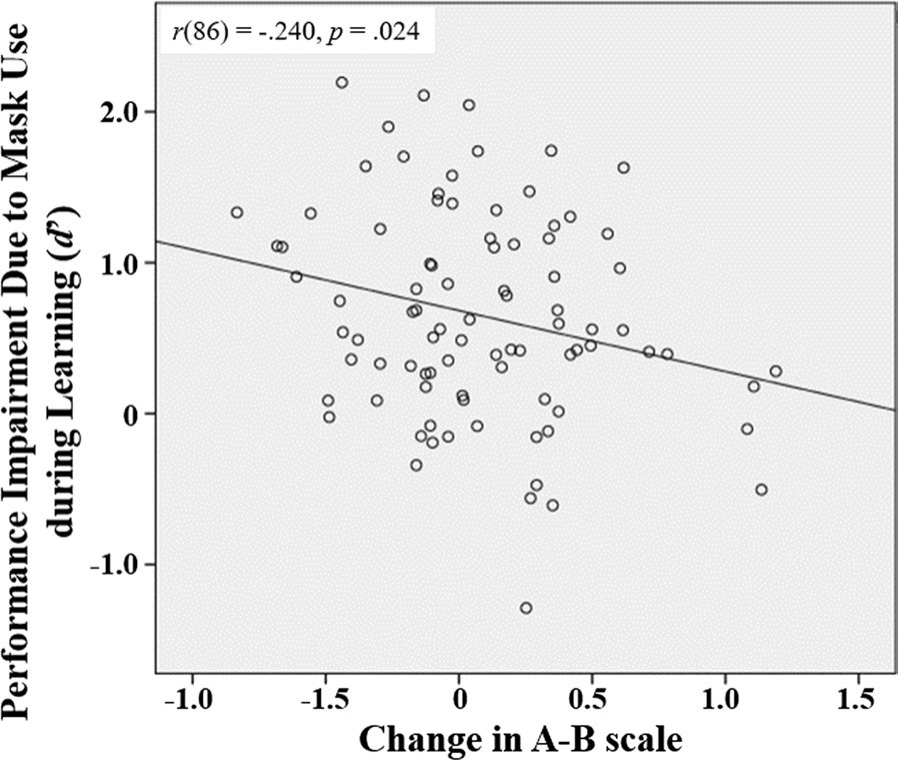
Correlation between the change in *A*–*B* scale due to mask use during learning (unmasked–unmasked condition minus masked–unmasked condition; more positive change indicates larger change toward the more eyes-focused Pattern *B* with mask use) and the corresponding performance impairment in *d*′: The more change toward the more eyes-focused Pattern *B*, the less the performance impairment

We then examined whether eye movement behavior change was associated with participants’ recognition performance change due to mask use during face recognition. On the change of recognition performance due to mask use during learning (unmasked–unmasked condition minus masked–unmasked condition), larger performance impairment in *d*′ was correlated with the smaller change toward Pattern *B* with mask use, *r*(86) =  − .240, *p* = .024 (Fig. 4). This correlation was still significant when we partialled out general intelligence as measured in RSPM, *r*(85) =  − .249, *p* = .020, or when we partialled out both general intelligence and cognitive ability measures, *r*(75) =  − .269, *p* = .018, using partial correlation analysis. These results suggested that individuals who adjusted their eye movement patterns to be more eyes-focused (Pattern *B*) when recognizing an unmasked face that was learned with a mask on during learning had less recognition performance impairment.

**Fig. 5:**
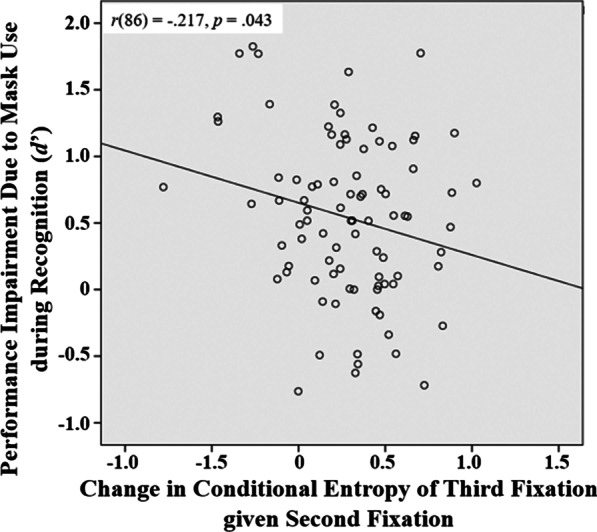
Correlation between the change in conditional entropy of the third fixation given the second fixation due to mask use during recognition (unmasked–unmasked condition minus unmasked–masked condition; more positive change indicates larger change toward lower entropy and thus more consistent transition with mask use) and the corresponding performance impairment in *d*′: The more change toward low-entropy/more consistent gaze transition behavior, the less the performance impairment

On the change of recognition performance due to mask use during recognition (unmasked–unmasked condition minus unmasked–masked condition), smaller performance impairment in *d*′ was correlated with larger change toward low conditional entropy of the third fixation given the second fixation with mask use, *r*(86) =  − .217, *p* = .043 (Fig. 5). This correlation was still significant when we partialled out general intelligence as measured in RSPM, *r*(85) =  − .235, *p* = .028, and it became marginal when we partialled out both general intelligence and cognitive ability measures, *r*(75) =  − .217, *p* = .058. In an explorative analysis examining which cognitive abilities were correlated with change in conditional entropy of the third fixation given the second fixation, we found that it was correlated with general intelligence (RSPM), *r*(86) =  − .276, *p* = .009, and TOL execution time, *r*(85) = .214, *p* = .045, suggesting that higher general intelligence and shorter execution time in TOL were associated with larger change toward low conditional entropy of the third fixation given the second fixation. These results suggested that individuals who had increased eye gaze transition consistency when recognizing a masked face that was learned without a mask on had less recognition performance impairment due to the mask use.

On the change in performance due to mask use in the face recognition task (unmasked–unmasked condition minus masked–masked condition), the change in face recognition performance was not associated with the change in any of the eye movement behavior measures.

## Discussion

Here, we examined how mask use affects performance and eye movement behavior in face recognition under three scenarios: seeing masked faces during face learning only, during face recognition only, and during both face learning and recognition. We tested the hypothesis that mask use impaired face recognition performance in all three scenarios, and the largest impairment may be found when the mask conditions between face learning and face recognition did not match. In these scenarios, individuals who could adjust their eye movement behavior according to the mask condition difference between face learning and recognition may have better performance. Consistent with our hypothesis, we found that as compared with the baseline condition where regular, unmasked faces were presented during both face learning and recognition, participants’ recognition performance was impaired in all three scenarios, with the largest impairment observed when there was a mismatch in mask condition between face learning and recognition. This finding is consistent with the encoding specificity effect reported in the literature (Uner & Roediger, 2018; Unsworth et al., 2020): A mismatch between information used for memory encoding and retrieval impairs memory recall performance. This is also consistent with the part-whole effect reported in the face perception literature, which is argued to be an indication of holistic face processing: People have better identification performance when a facial part (e.g., the eyes) is presented in the context of the whole face than when it is presented in isolation (after face learning with the whole face; Seitz, 2001; Tanaka & Farah, 1993).

In addition, we found that when recognizing a regular, unmasked face, although participants performed worse when the face was learned with a mask on than without, whether the face was learned with or without a mask on did not significantly change participants’ eye movement pattern or consistency. This result suggested that eye movements during recognition are mainly driven by the information available during recognition but not that during learning (Chuk et al., 2017a). While this result also suggested that recognition performance impairment due to mask use during face learning could not be accounted for by change in eye movement pattern or consistency during recognition at the group level, an interesting individual difference effect was found: As compared with the baseline scenario where unmasked faces were used during both face learning and recognition, the recognition performance impairment due to mask use during face learning could be predicted by the amount of eye movement change toward a more eyes-focused pattern during recognition (Patten *B* in Fig. 3a): The larger the change in eye movement pattern toward a more eyes-focused pattern, the less the recognition performance impairment due to mask use during face learning (Fig. 4). This association remained significant after general intelligence and cognitive ability factors were accounted for, suggesting that the contribution from eye movement pattern change was unlikely to be confounded with general intelligence or eye movement pattern.

In the literature on visual cognition, it has been proposed that the mental representation of a visual object or pattern includes the perceptuomotor cycle involved during memory encoding (i.e., the scan path theory; Noton & Stark, 1971a, 1971b). Thus, the eye movement pattern elicited during memory encoding may become part of the mental representation, which may be reactivated and affect the viewer’s eye movement pattern and performance during memory retrieval (e.g., Blais et al., 2008; Laeng & Teodorescu, 2002). For highly learned skills such as face recognition, this perceptuomotor memory during face learning may interact with the visual routine memory developed through years of experience to affect eye movement planning behavior during recognition. Chuk et al. (2017a) showed that in face recognition, the match between eye movement pattern adopted during face learning and recognition did not predict recognition performance. In contrast, a more eyes-focused eye movement pattern during recognition predicted better recognition performance. This result suggests that retrieving diagnostic features plays a more important role in successful recognition than reactivating perceptuomotor memory during the recognition of unmasked faces that were also learned without a mask on. In contrast to this finding, here we showed that when there is a mismatch between the information available for memory encoding and retrieval due to mask use, as in the case of recognizing an unmasked face that was learned with a mask on, the reactivation of perceptuomotor memory may become beneficial, as it helps the viewer to attend to the features that were available during memory encoding when these features are presented in a different context during retrieval.

We also found that when recognizing masked faces, participants had more eyes-focused eye movement pattern (Pattern *B* in Fig. 3a) and more consistent gaze transition behavior (as reflected in the conditional entropy of the early fixations in a trial) than recognizing unmasked faces, regardless of whether the faces were learned with or without a mask on. The task demands of recognizing a masked face differ from our usual experience of recognizing unmasked faces. Thus, new visual routines have to be developed to solve the new recognition problem. Our results suggested that adopting a more eyes-focused eye movement pattern and engaging in more consistent gaze transition behavior when recognizing masked faces as compared with recognizing unmasked faces (regardless of whether faces were learned with or without a mask on) were a general strategy adopted by the participants for solving this new recognition problem. A more eyes-focused pattern facilitates information extraction from the face region not covered by the mask, whereas more consistent gaze transition behavior may be related to a more restricted region (i.e., only the forehead and eye regions are available for recognition) available for developing a visual routine, leading to more predictable gaze transition behavior.

In addition, we observed an interesting individual difference effect: As compared with the baseline condition where there was no mask during both face learning and recognition, the recognition performance impairment due to mask use during recognition could be predicted by the amount of change toward more consistent gaze transition behavior (conditional entropy of the third fixation given the second fixation; Fig. 5): The larger the change in eye movement consistency toward more consistent transition behavior, the less the recognition performance impairment due to mask use during face recognition. This correlation remained significant after the contribution from general intelligence was partialled out and became marginal when both general intelligence and cognitive ability factors were partialled out. This result suggested that this association was unlikely to be confounded with general intelligence, although it may be related to some cognitive abilities. Indeed, in an explorative analysis, we found that this change in consistency of gaze transition behavior was significantly correlated with participants’ general intelligence and TOL performance. Individual differences in gaze transition consistency when solving a recognition problem typically reflect differences in the ability to achieve efficient and successful task processing: Those who are able to discover diagnostic information and learn to identify it efficiently and effectively would develop a more consistent visual routine that leads to better performance. For example, young children who have more consistent eye movement patterns across trials when learning to recognize faces are shown to have better recognition performance than those with less consistent patterns (Hsiao et al., 2020). Thus, change in gaze transition consistency may reflect the ability to discover and identify diagnostic features for recognizing masked faces. Note, however, that this relationship between change in gaze transition consistency and change in recognition performance was only observed when the mask conditions during face learning and recognition did not match, i.e., recognizing masked faces that were learned without a mask on, but not when recognizing masked faces that were also learned with a mask on. Thus, this gaze transition consistency effect may be related to the ability to develop a new visual routine when only part of the information learned during memory encoding (face learning) is available for retrieval during recognition.

Together the findings in the current study suggest that when the mask condition differs between face learning and recognition, it presents a particularly challenging scenario where face recognition performance can be significantly affected. In this scenario, individuals who have better ability to adjust their eye movement strategy according to the mask condition difference are affected less by mask use and thus may have a better ability to adapt to life in a society with widespread mask use brought about by the COVID-19 pandemic. This finding has important implications for identifying individuals vulnerable to the impact of mask use and potential remedial strategies. More specifically, the ability to adjust eye movement strategies according to mask conditions during face recognition may require good cognitive flexibility to switch between perceptuomotor and visual routine memories, and good problem-solving skills to develop new visual routines when there is a mismatch between information available for memory encoding and retrieval. Thus, it may be particularly challenging to young children, whose cognitive flexibility and problem-solving skills are still developing (e.g., Peng et al., 2018); older adults, whose cognitive abilities may have declined (e.g., Giller & Beste, 2019); and individuals with autism spectrum disorders (ASD), who are typically characterized by low cognitive flexibility (e.g., English et al., 2017; Fujino et al., 2019; Soriano et al., 2018). Explicit instructions on strategies that may benefit recognition performance, such as adjusting to a more eyes-focused eye movement pattern when recognizing an unmasked face that was learned with a mask on, may help these vulnerable individuals adapt better. Future work will examine these possibilities.

## Conclusions

In conclusion, here we showed that while mask use impairs face recognition performance regardless of whether masks are used during face learning only, during face recognition only, or during both face learning and recognition, the largest impairment happens when there is a mismatch between the mask conditions during face learning and recognition. When recognizing regular, unmasked faces that are learned with a mask on, those who adjust their eye movement strategy to be more eyes-focused such that it is more consistent with their perceptuomotor memory during face learning are less affected by mask use in recognition performance. When recognizing masked faces that are learned without a mask on, those who demonstrated more consistent gaze transition behavior, an indication of better visual routine development ability, are less affected by mask use in recognition performance. These results suggest that when there is a mismatch between the mask conditions during face learning and recognition, those who have a better ability to adjust their information extraction strategy according to the mask condition difference are affected less by mask use. This finding has important implications for identifying individuals who may have poor cognitive flexibility or problem-solving skills to adjust eye movement strategy and thus may be vulnerable to the impact of mask use on face recognition performance, including children, older adults, and individuals with ASD, and the possibility to provide in-time intervention for them.

## Data Availability

The datasets generated and/or analyzed during the current study are available in the OSF data repository, https://osf.io/g6es5/?view_only=5904749c5e124e0b81cde7feb071bd00.

## Abbreviations

ASD: Autism spectrum disorder
EMHMM: Eye movement analysis with hidden Markov models
RT: Reaction time
RSPM: Raven’s Standard Progressive Matrices
TOL: Tower of London
WM: Working memory
ROIs: Regions of interest
VBEM: Variational Bayesian expectation–maximization
VHEM: Variational hierarchical expectation–maximization

## Footnotes

1 Note that in reporting confidence interval information, as suggested by Lakens (2013), a 90% CI was used for ANOVA results and a 95% CI was used for *t* test results due to the fact that the *F* test in ANOVA is usually a one-sided test, and adopting a 95% CI may include 0 in the interval when the test is statistically significant.
